# The Research Progress of Direct KRAS G12C Mutation Inhibitors

**DOI:** 10.3389/pore.2021.631095

**Published:** 2021-04-23

**Authors:** Ai Yang, Min Li, Mingzhi Fang

**Affiliations:** Department of Oncology, Nanjing Hospital of Chinese Medicine Affiliated to Nanjing University of Chinese Medicine, Nanjing, China

**Keywords:** KRAS mutation, targeted drugs, oncogene, inhibitor, oncology

## Abstract

**Abstract:** KRAS mutations have long been considered undruggable. However, a series of direct KRAS mutation inhibitors have been developed since the switch II pocket was discovered recently. This review will summarize progress in the development of direct KRAS G12C mutation inhibitors, current relevant drugs under study and challenges that need to be considered in future research.

## Research background

### Explorations of RAS Mutations

**FIGURE 1:**
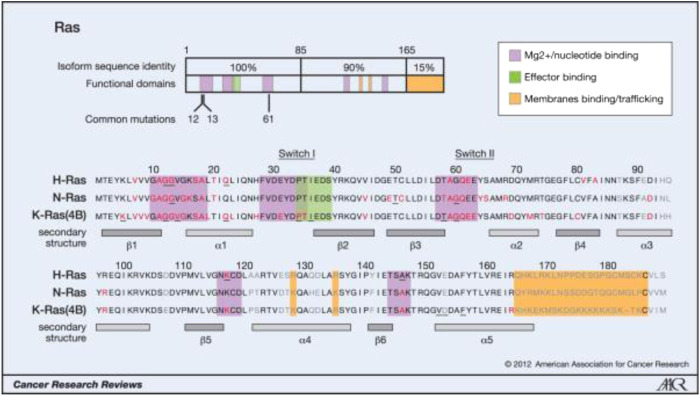
Oncogenic mutations of Ras isoforms.

**FIGURE 2:**
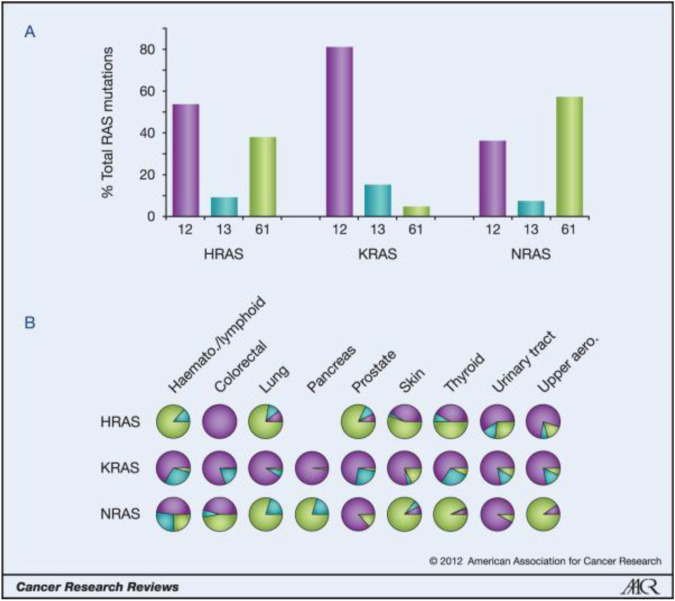
Ras isoform-specific codon mutation bias.

Weinberg’s laboratories found HRAS in the human bladder cancer cell line T24/EJ in 1982. this discovery made RAS the first human tumor gene to be identified. RAS mutations have been detected in bladder cancer, breast cancer, colon cancer, etc., in addition, they are the most common mutations in tumors, being found in approximately 30% of human tumors. The Ras protein family includes three subtypes (KRAS, HRAS, and NRAS), and 85% of Ras-driven cancers are caused by KRAS mutations [1]. Most RAS mutations (98%) affect G12, G13, and Q61. KRAS mutations are the most common in solid cancers, such as pancreatic cancer, colorectal cancer and, lung cancer, and in these cancers, the mutations affect G12. NRAS mutations are the most frequent in melanoma and myeloid leukemia, and most of the mutations affect Q61. HRAS mutations are the most common in bladder cancer, and the mutations affect G12 and Q61 [2]. For more information, please see Figure 1 and Figure 2.

The key oncogenic mutations occur in the region that is shared between the three isoforms. Forty-four separate point mutations have been characterized in Ras isoforms, with 99.2% of all mutations occurring in codons 12, 13, and 61. The mutations cluster in and around loops 1, 2, and 4, which are responsible for nucleotide binding, and result in enhanced GTP binding. The residues mutated in cancer are shown in red, those mutated in developmental disorders are underlined, and residues that vary between isoforms are shown in gray (26, 65, 66).

A) K-Ras is typically mutated at codon 12, whereas N-Ras is typically mutated at codon 61. H-Ras shows mutations in both codons. The data are averages of percentages collated from all cancers with at least 20 tumors scored. B) An analysis of individual cancer types revealed isoform-specific patterns of codon mutations even within the same tissue. Pie chart colors–black: codon 12; gray: codon 13; white: codon 61.

### Drug Resistance Mechanism of RAS Mutation

**FIGURE 3:**
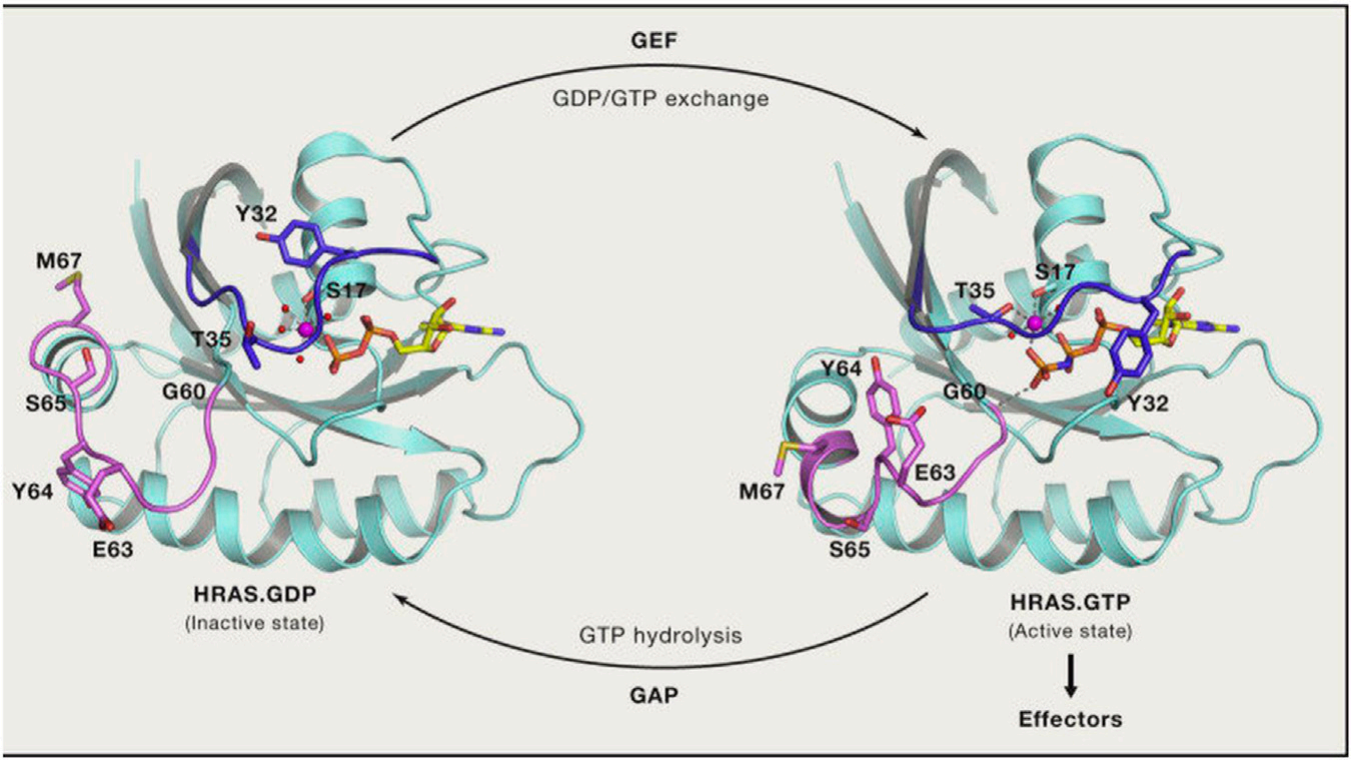
RAS GTP/GDP change.

In the normal signal transduction pathway, RAS proteins are located on the inner side of the plasma membrane, where they interact with GDP to remain in an inactive state. Loading of GTP through guanine nucleotide exchange factors (GEFs; e.g., SOS1), triggers subsequent interactions with effector proteins that activate RAS-dependent signaling. Then, RAS proteins hydrolyze GTP to GDP through their intrinsic GTPase activity, which is dramatically enhanced by GTPase-activating proteins (GAPs). For additional details, see Figure 3.

However, mutations at codons 12 and 13 in RAS proteins impair GAP-stimulated GTP hydrolysis, leaving RAS predominantly in the GTP-bound state, the active state [3]. For many years, drugs targeting KRAS directly did not exist because of its small size, smooth surface, and strong binding affinity for GTP (with KRAS able to bind with GTP at picomolar concentrations) [4, 5].

### Research of Related Drugs

Currently, there are three strategies for targeting KRAS mutation. One is to develop drugs that can compete with GTP. However, we found two problems with this strategy in our study: the first problem is the high affinity between GTP and KRAS, the second is the high concentration of GTP in cells *in vivo* (as well as the low specific binding of GTP analogs to KRAS) [6]. Another strategy is to prevent KRAS from combining with GEFs, which leads to the activation of KRAS. For example, Burns MC et al [7] found a small molecule that can combine with SOS1, which provided a basis for Xu L et al [8] to find related inhibitors. The third strategy is to change the location of the KRAS protein. The key to this strategy is PDEδ, which inhibits the activation of the ERK and KRAS signaling pathways. Currently, some groups are preparing to develop related small-molecule drugs [9].

## Early Studies

**FIGURE 4:**
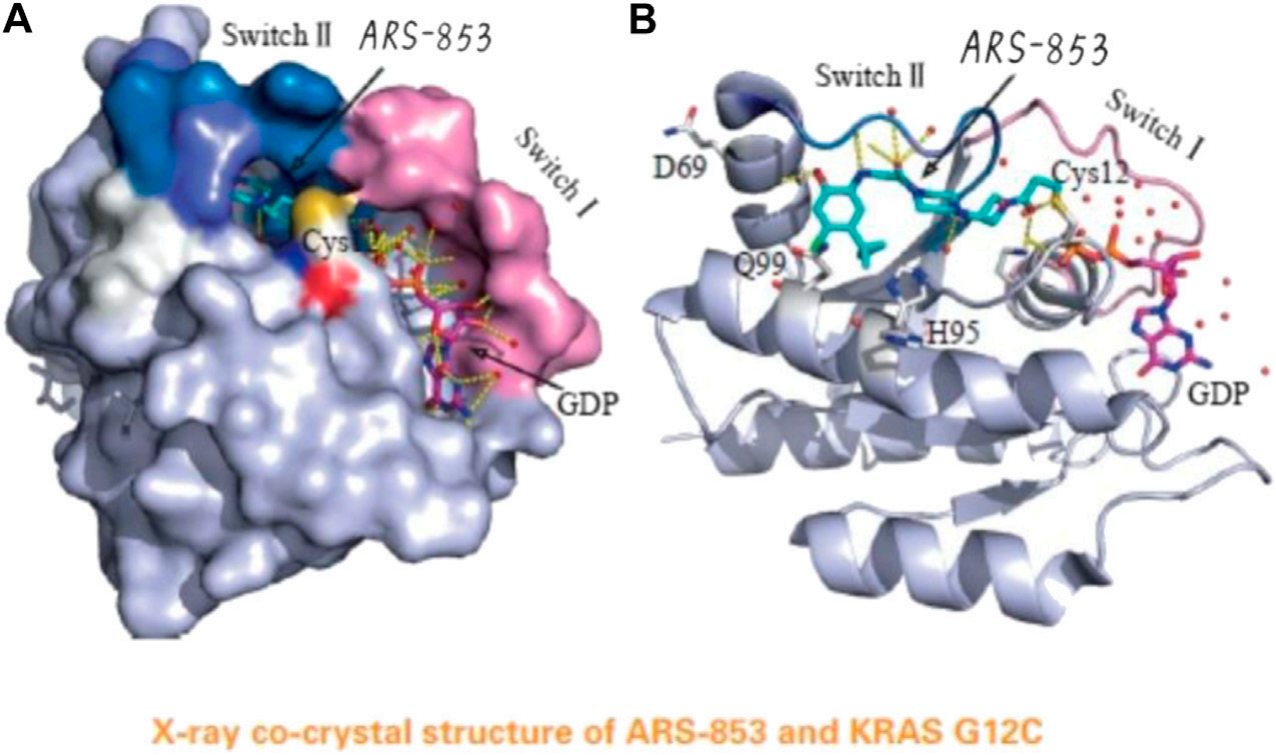
The acrylamide of ARS-853 can form covalent bond with 12 cysteine and extend to switch Ⅱ region, aromatic nucleus occupies hydrophobic region, the cyclopropyl group and the surrounding amino acids form a strong van der Waals force.

In 2013, Shoket and his colleagues [10] proposed the idea of targeting the 12th cysteine of mutant KRAS. They set up a screening model of disulfide fragments based on the structure of KRAS G12C in the GTP-bound state and identified 2E07(18) and 6H05(19), both of which bind to the switch II pocket. This finding laid the foundation for further research. In 2014, Lim et al [11] proposed the molecule SML-10-70-1 in a paper published in Angewandte Chemie; SML-10-70-1 was able to achieve a small decrease in pERK and pAkt at 100 μM and had no effect on wild-type KRAS. However, it also affected KRAS G12 S mutant cells, suggesting that it has off-target effects. Lito’s group [12] further investigated a KRAS G12C covalent binding inhibitor called ARS-853 (See Figure 4). They found that ARS-853 could only interact with KRAS G12C in the GDP-bound state by comparing the therapeutic effect of ARS-853 on cells with KRAS G12C in the GTP-bound state or GDP-bound state. The nucleotide state of KRAS G12C is in dynamic flux, and ARS-853 fully integrates with the protein and inhibit its activation by modulating the upstream signaling factor [13, 14]. The disadvantages of ARS-853 are its poor stability in plasma (t^1/2^ < 20 min) and its low bioavailability after oral administration in mice (F < 2%). As such, ARS-853 has not been further studied.

## Current Research

### ARS-1620

#### The Mechanism of Action of ARS-1620

**FIGURE 5:**
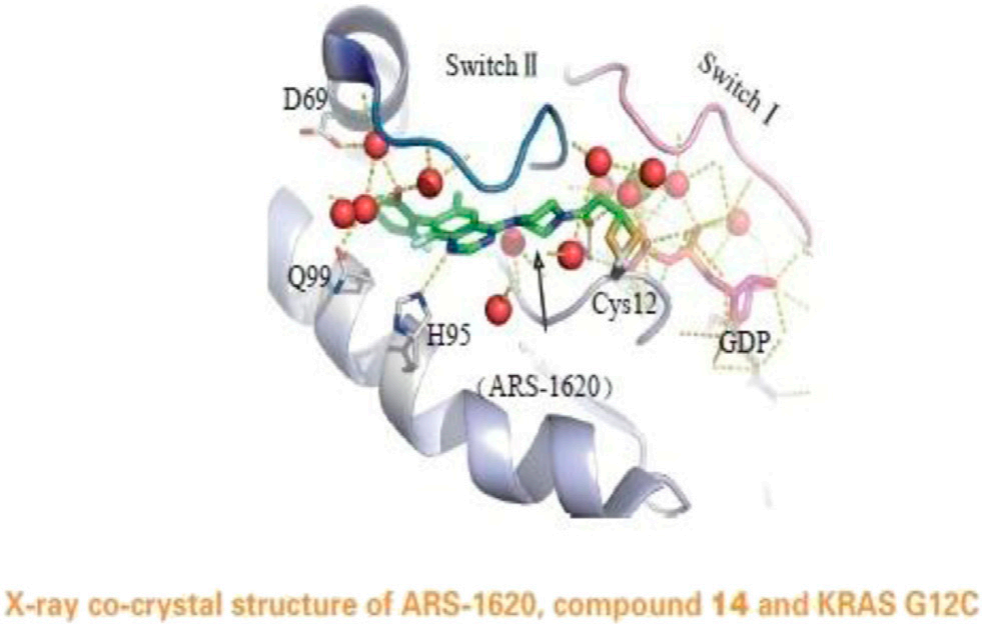
X-ray co-crystal structure of ARS-1620,compound 14 and KRAS G12C.

After improving the core structure of ARS-853, Janes et al [15] synthesized a quinazoline core as a versatile lead scaffold. Among the drugs using this scaffold, the representative drug is ARS-1620 (See Figure 5). ARS-1620 covalently modified KRAS G12C at a rate of 1,100 ± 200 M^−1^s^−1^ (kobs/[I]), 10 times higher than the rate of ARS-853. In H358 cell lines, ARS-1620 inhibited RAS-GTP binding and the phosphorylation of MEK, ERK, RSK, S6, and AKT in a dose-dependent and selective manner but had no effect on the negative control group (A549, H460, and H44 cells), indicating that it selectively inhibited G12C mutant cells but not wild-type cells. Moreover, ARS-1620 showed excellent oral bioavailability (F >60%) and had sufficient plasma stability in mice. To further determine whether ARS-1620 has sufficient covalent and pharmacokinetic properties to enable it to function *in vivo*, the researchers administered ARS-1620 to a subcutaneous xenograft model with KRAS G12C-bearing tumors at a dose of 200 mg/kg/day and found that the tumor obviously receded.

#### Combination Therapies Including ARS-1620

Using ARS-1620 alone confers drug resistance to tumor cells and leads to tumor recurrence after regression. Miriam et al [16] found that deletion of MTOR genes can make cells significantly sensitive to KRAS and IGF1R inhibitors. The researchers also found that blocking the IGF1R, MAPK, and PI3K/AKT/mTOR signaling pathways simultaneously led to the death of cancer cells carrying KRAS mutations. On the basis of previous findings, they screened the entire genome (16,019 genes) in shRNA-infected KRAS-mutant non-small-cell lung cancer (NSCLC) H23 cells, trying to identify triple combination therapies using ARS-1620, mTOR inhibitors and the IGF1R inhibitor linsitinib. They found that triple therapy could significantly reduce tumor size in mice and humans, and cell growth was not detected for 17 days. Signaling pathway analysis showed not only that ARS-1620 inhibited ERK and S6 phosphorylation but also that the triple therapy persistently inhibited AKT and S6 phosphorylation. In addition, the triple therapy reduced the reactivation of RAS and ERK activation for 48 h.

### AMG-510

#### The Mechanism of Action of AMG-510

**FIGURE 6:**
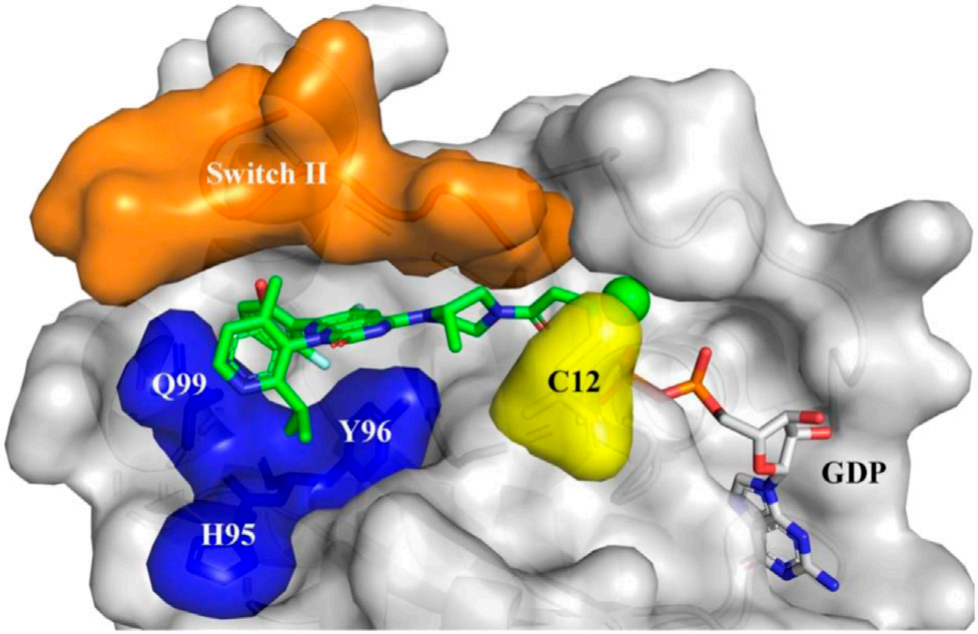
Sotorasib (AMG510) binding to KRAS-G12C protein. Orange: Switch II domain; Yellow: C12 residual; Blue: A cryptic pocket composed of H95, Y96, and Q99. AMG510 (green) and GDP (gray) are shown in sticks.

Due to the small size of the switch II pocket, additional protein-ligand interactions are limited; this limitation makes further improvements in ARS-1620 difficult. However, with the discovery of the obvious hydrogen bonding between ARS-1620 and His95, new progress has been achieved by researchers. A surface groove formed by the alternative orientation of His95 can be occupied by aromatic rings, and this discovery might enhance the interaction between drugs and KRAS G12C [17]. Therefore, a new drug called AMG-510 (See Figure 6), which has similar ligand structure to ARS-1620, was developed by Cannon et al. [18] However, His95 is a newly identified site for AMG-510 binding. AMG-510 almost completely inhibited pERK (IC50 ≈ 0.03 μM) in NCI-H358 and MIA PaCa-2 cells after 2 h of treatment, and its IC50 value was 20 times that of ARS-1620. In terms of cell viability, AMG-510 decreased the viability of NCI-H358 and MIA PaCa-2 cells (IC50 ≈ 0.006 μm and 0.009 μm, respectively), with IC50 values approximately 40 times those of ARS-1620. However, the strong binding affinity of AMG-510 may covalently link it to other cysteine-containing proteins, which is a problem that needs to be addressed in the clinical trial stage [19].

#### Clinical Application of AMG-510

In a patient trial (four patients with NSCLC), the researchers found that treatment with AMG-510 resulted in objective partial responses (PRs) in two patients and stable disease (SD) in two patients. The two patients with a PR had documented disease progression on treatments including carboplatin, pemetrexed, and nivolumab. After 6 weeks of AMG-510 treatment, the first group (180 mg) exhibited tumor shrinkage of 34%, and the second (360 mg) exhibited tumor shrinkage of 67%. A computed tomography (CT) scan 18 weeks later showed that the second group of tumors had shrunk to the point at which they were no longer visible. Amgen [20] presented preclinical data of AMG-510 at the American Association for Cancer Research (AACR) meeting in 2019. They used homologous tumor cell lines *in vitro* that were suitable for testing AMG-510, checkpoint inhibitors, and AMG-510 combined with checkpoint inhibitors and ultimately found that AMG-510 could clear colon cancer cells from mice. In the phase I/II study [21, 22], a total of 35 patients were recruited into four low-dose groups (180, 360, 720, and 960 mg). The outcome was evaluated in 29 patients, 10 of whom had NSCLC (most of them had previously received ≥3 kinds of treatments). The results showed that half of the NSCLC patients had objective PRs. The most common treatment-related adverse events (AEs) were loss of appetite, diarrhea, fatigue, headache, cough, hot flashes, and nausea. The severe adverse events (AEs) in 6 patients, including grade III pneumonia, malignant biliary obstruction, and grade IV pericardial effusion, were all considered to be AMG-510 related. A recent study, presented in September at the 2019 World Lung Cancer Congress meeting, covered 34 NSCLC patients who received AMG-510 treatment; 19 of them were treated at a dose of 180 mg, and 15 of them had stage II disease and received a dose of 960 mg; no dose-limiting toxicity was observed. Of the 23 patients who received a CT scan for 6 weeks or had early progressive disease, the objective response rate was 48%, and the disease control rate was 96%. Among the evaluable patients, 13 received 960 mg of phase II treatment, 7 people (54%) achieved PR, and 6 people (46%) achieved SD.

#### AMG-510 Combination Therapy

The clinical results of strategies combining BRAF and MEK inhibitors [23] suggest that combinations with AMG-510 and other inhibitors may show enhanced tumor cell killing effects and overcome drug resistance. One team conducted experiments *in vitro* in KRAS G12C cell lines using AMG-510 and HER kinase, EGFR, SHP2, PI3K, AKT, and MEK inhibitors and found that the combination of AMG-510 and MEK inhibitors significantly enhanced antitumor activity. In an AMG-510 monotherapy mouse experiment, the tumors of mice lacking T cells were likely to recur. The researchers used a model called CT-26KRASG12C [24, 25], which is widely used to assess the effectiveness of immunotherapy and combinations of immunotherapy and targeted drugs, to assess AMG-510 combined with a PD-1 inhibitor. As a result, 1/10 of the tumors completely disappeared when AMG-510 or the PD-1 inhibitor was used alone. However, 9/10 of the tumors disappeared completely with the combination therapy. Treatment was stopped after the 43rd day, and no recurrence was seen in any complete responder after 112 days.

### MRTX849

#### The Mechanism of Action of MRTX849

**FIGURE 7:**
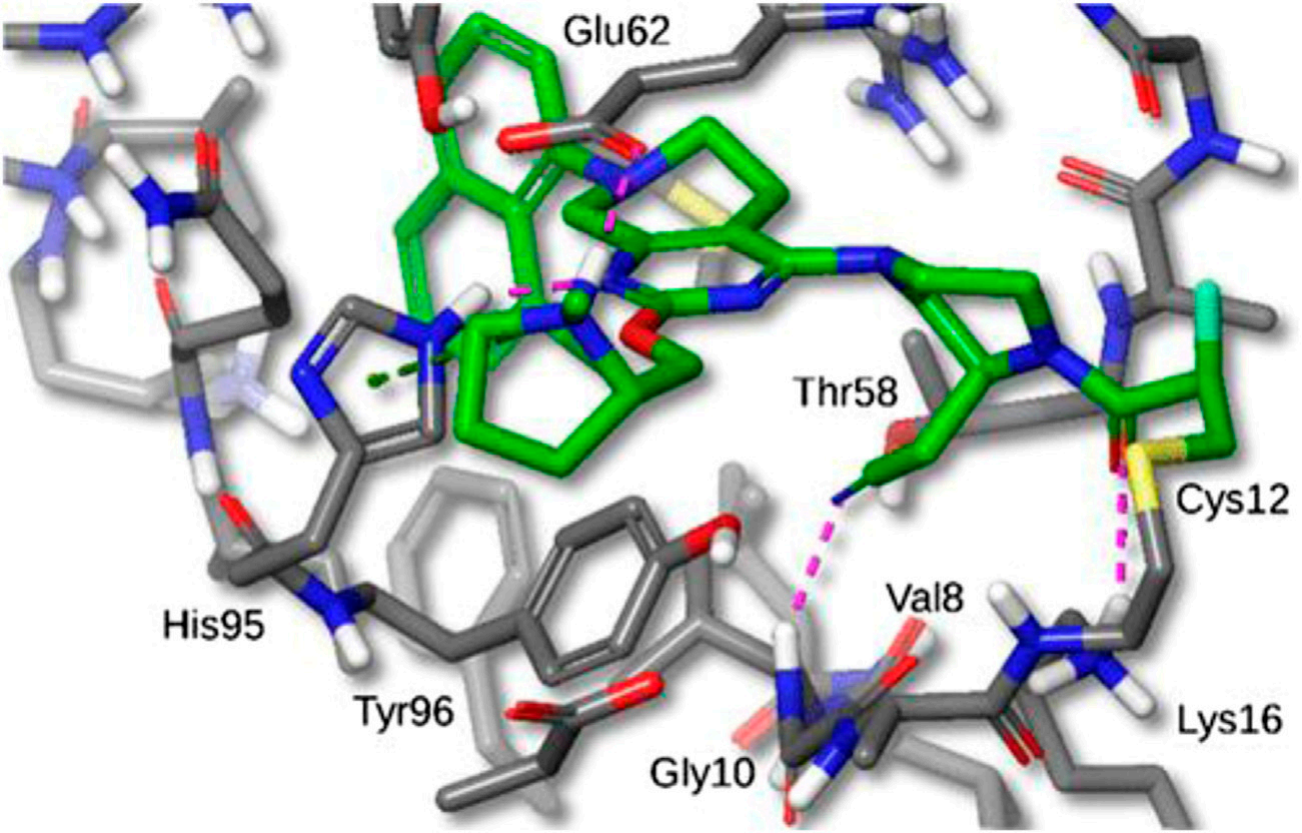
X-ray crystal structure of MRTX849 bound to KRASG12C with 1.94 Å resolution, hydrogens added for clarity.

Hallin et al [26] discovered MRTX849 (See Figure 7) while performing favorable drug optimization experiments. Through LC/MS-based tests of KRAS G12C protein modification, they found that MRTX849 also combined with GDP-bound KRAS G12C and locked it in the inactive state. In addition, MRTX849 also inhibited KRAS-dependent signal transduction effects, including ERK1/2 phosphorylation, S6 phosphorylation, and the expression of ERK-regulated DUSP6, and the greatest effect was observed at 24 h. The half-life was approximately 25 h after a single dose. To assess the pharmacodynamics of MRTX849, the researchers fed mice H358 xenotransplantation by gavage. The dose of MRTX849 found to covalently modify KRAS G12C was 30 mg/kg, and the effects increased in a dose-dependent manner; the maximum effective dose of MRTX849 was between 30 and 100 mg/kg/day. The researchers performed experiments with a dose of 100 mg/kg/day in cell line-derived xenograft (CDX) and patient-derived xenograft (PDX) models. After approximately 3 weeks of treatment, 17 of the 26 models (65%) showed tumor regression over 30%.

#### Clinical Application of MRTX849

The preliminary results of a phase I/II trial, which began in January 2019, were first presented at the AACR-NCI-EORTC Conference in 2019 (International Conference on Molecular Targets and Cancer Therapy) [27]. Of the 12 assessable patients (6 with NSCLC, 4 with colorectal cancer (CRC) and 2 with appendix cancer), 3 (50%) NSCLC patients and 1 (25%) CRC patient achieved a PR. The most common adverse events from MRTX849 were grade 1/2 diarrhea or nausea, and no other serious adverse reactions have yet been found.

#### MRTX849 Combination Therapy

MRTX849 combined with HER family inhibitors synergistically inhibited tumor cell viability in most cell lines and was first tested in combined screening *in vitro*. The researchers selected afatinib as a representative HER family inhibitor and chose five tumor models (KYSE410, SW1573, H2122, H2030, and LU6405 cells) with partial sensitivity to single-agent MRTX849 or refractoriness to the treatment, and all the cell lines showed obvious antitumor activity. They found that HER family activation *in vivo* may limit MRTX849-mediated inhibition of the ERK and mTOR-S6 signaling pathways, while administration of afatinib combined with MRTX849 could limit feedback reactivation of ERK signaling and showed complementary inhibition of AKT-mTOR-S6 signaling, significantly enhancing antitumor activity. Moreover, the combination of SHP2 inhibitors and MRTX849 also achieved inhibition of tumor activity. Administration of the mTOR inhibitor vistusertib with MRTX849 induced complementary inhibition of the ERK and mTOR-S6 signaling pathways, showing extensive antitumor activity.

### BI-2852

**FIGURE 8:**
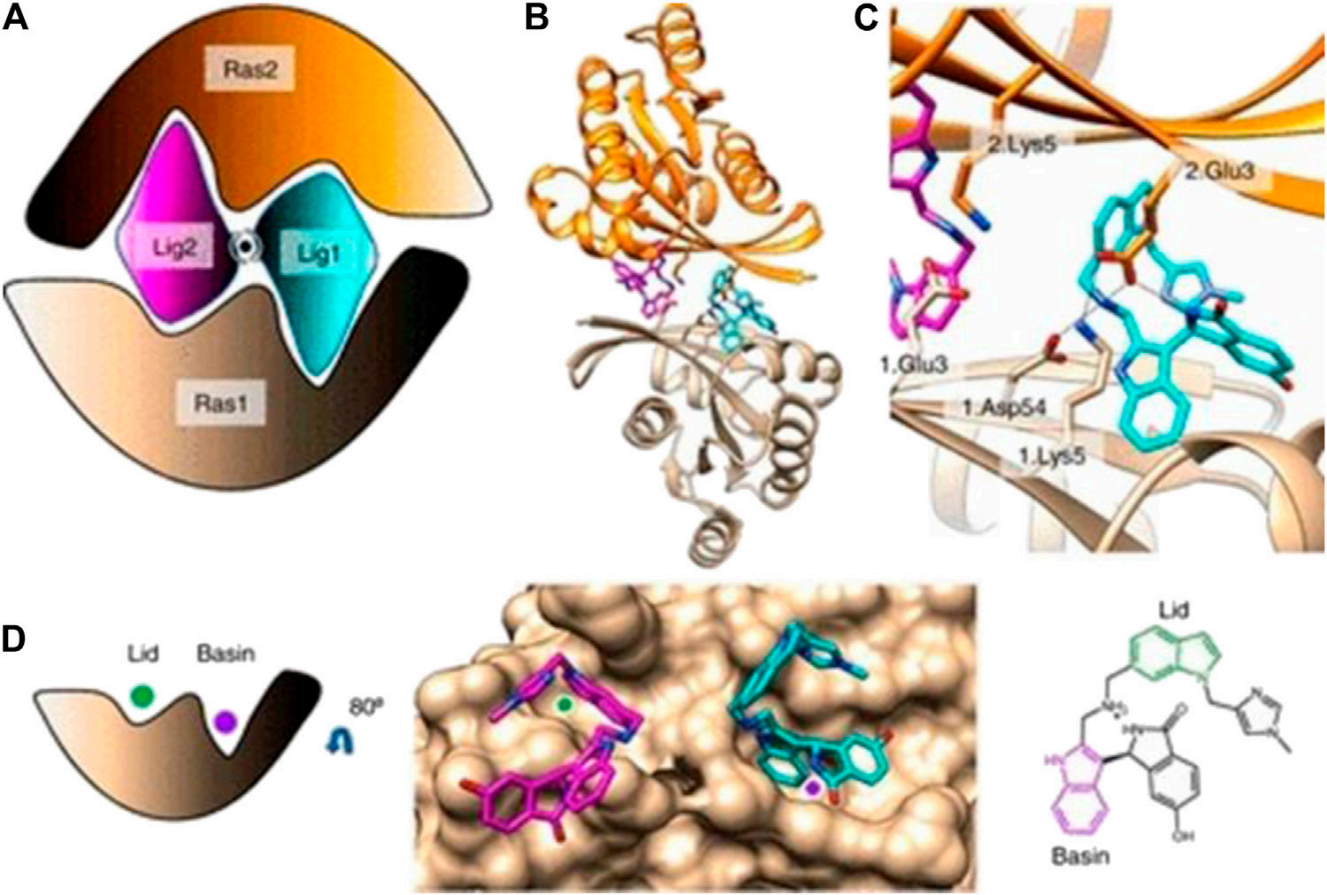
**(A)** Cartoon of BI-2852 KRAS dimer. Lig1 (cyan) and Lig2 (magenta) interact with both Ras1 (beige) and Ras2 (orange). **(B)** KRAS (ribbons) and key side chains are shown. **(C)** Zoom-in of the binding site: salt–bridge interactions shown (dashed lines). **(D)** Lid (green) and Basin (purple) are indicated on Ras1. Both Lig1 and Lig2 are shown on Ras1. The indole rings, colored green and purple, engage the Lid and Basin, respectively.

Kessler et al [28] developed BI-2852 (See Figure 8), which is different from the drugs mentioned above; it binds to the RAS switch I/II pocket with nanomolar-level affinity (recent rabbit experiments [29, 30] have found that it modulates GEFs, GAPs and downstream effector interactions). In addition, BI-2852 can interact with KRAS in both the GDP- and GTP-bound states, which in turn blocks interactions of all GEFs, GAPs and effectors with KRAS and prevents the propagation of downstream signaling pathways and KRAS proliferation.

**TABLE 1:** Summary table (include Drug name, Mechanism of action, Combination therapy, Clinical trial stage, Clinical effect, Trial ID).

	Mechanism of action	Combination therapy	Clinical trial stage	Clinical effect	Trial ID
ARS-853	Selective binding with RAS in GDP-bound state	—	—	—	—
ARS-1620	Selective binding with RAS in GDP-bound state	Triple therapy of ARS-1620, an mTOR inhibitor and the IGF1R inhibitor linsitinib	—	—	—
AMG-510	Binds with His95 with nanomolar-level affinity	①AMG-510 combined with a MEK inhibitor	Phase I/II	Half of the assessable patients achieved objective partial responses	NCT03600883
		②AMG-510 combined with a PD-1 inhibitor		
MRTX849	Selective binding with RAS-GDP, maintaining it in an inactive state	①MRTX849 combined with the HER family inhibitor afatinib	Phase I/II	—	NCT03785249
		②MRTX849 combined with an SHP2 inhibitor		
		③MRTX849 combined with the mTOR inhibitor vistusertib		
BI-2852	Binds with the switch II pocket regardless of GTP or GDP status	—	—	—	—
ARS-3248	Based on ARS-1620	—	Phase I	—	NCT04006301
LY3499446	—	—	—	—	NCT04165031
US 2019/0248767A1	—	—	—	—	—
WO 2019/110751 A1	—	—	—	—	—

### ARS-3248

Wellspring Biosciences and Janssen recently obtained approval for research of their new KRAS G12C inhibitor ARS-3248, which is based on ARS-1620. ARS-3248 will be tested in a phase 1 trial, and no research data have been published yet.

### Other New Drugs

LY3499446, tetrahydroquinazoline derivatives (US 2019/0248767A1) and tetracycline compounds (WO 2019/110751A1) are also currently in the R&D phase of development. Among the three, LY3499446 appears to be further in the development process, and a phase I study related to LY3499446 has begun to recruit patients in Australia. LY3499446 will be assessed as monotherapy in this study and used in combination with other drugs, including abemaciclib, cetuximab, and erlotinib, in advanced solid tumors (including NSCLC and CRC) [31].

## Challenges

KRAS inhibitors are in phase I clinical trials, and some early data have showed inactivity of some cancer cells after treatment, while others avoid this effect to restore proliferation. Lito et al [32] found that some KRAS receptor tyrosine kinases can trigger nucleotide exchange. AURK signal transduction promotes effector activation and cell cycle progression; cells with new KRAS G12C mutation can quickly escape inhibition and resume proliferation, and these cells can rapidly change to an active state that is insensitive to drugs. KRAS inhibitors are a new promising tool, and an increasing number of related drugs will be developed. However, most of these agents lack data to prove their clinical effectiveness, and their side effects are still unclear. We cannot yet determine whether KRAS G12C inhibitors can achieve maximum effects as monotherapy, and the current combinations including KRAS G12C inhibitors do not have guaranteed lasting efficacy. In addition, new challenges will likely be encountered in the future.
