# Corrigendum: The Research Progress of Direct KRAS G12C Mutation Inhibitors

**DOI:** 10.3389/pore.2021.1610250

**Published:** 2022-02-24

**Authors:** Ai Yang, Min Li, Mingzhi Fang

**Affiliations:** Department of Oncology, Nanjing Hospital of Chinese Medicine Affiliated to Nanjing University of Chinese Medicine, Nanjing, China

**Keywords:** KRAS mutation, targeted drugs, oncogene, inhibitor, oncology

In the original article, there were mistakes in the legend for all of the figures as published. The source of the pictures cited in the article were not included. The correct legends appear below.

**FIGURE 1:**
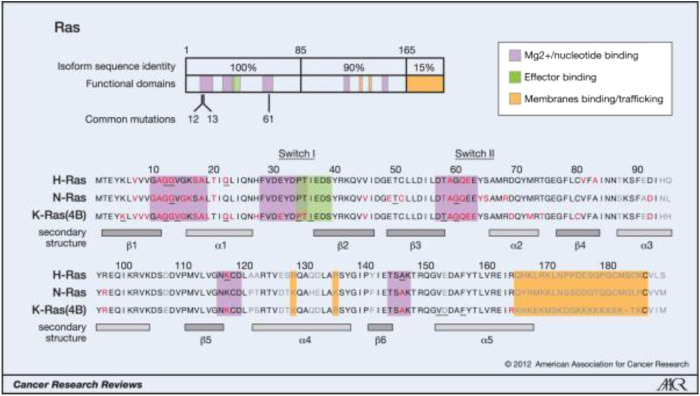
Oncogenic mutations of Ras isoforms. (Reprinted by permission from Cancer Research [3]).

**FIGURE 2:**
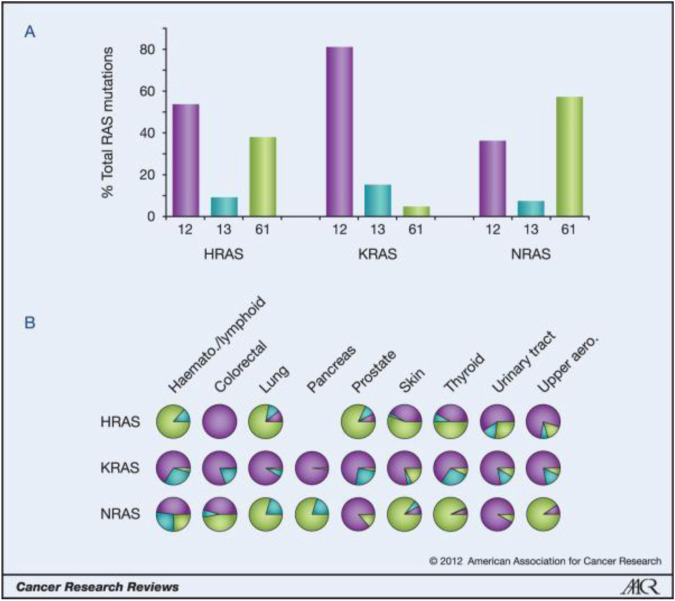
Ras isoform-specific codon mutation bias. (Reprinted by permission from Cancer Research [3]).

**FIGURE 3:**
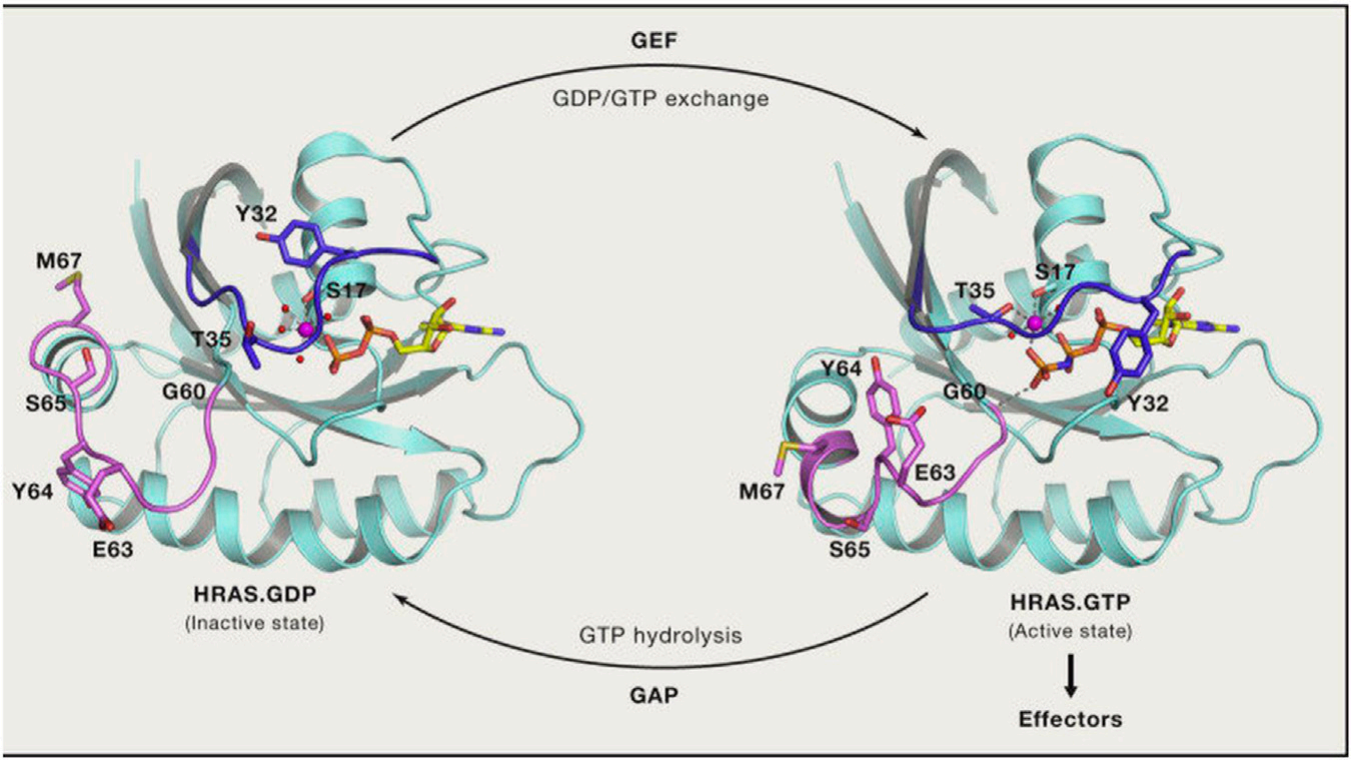
RAS GTP/GDP change. (Reprinted by permission from Cell [4]).

**FIGURE 4:**
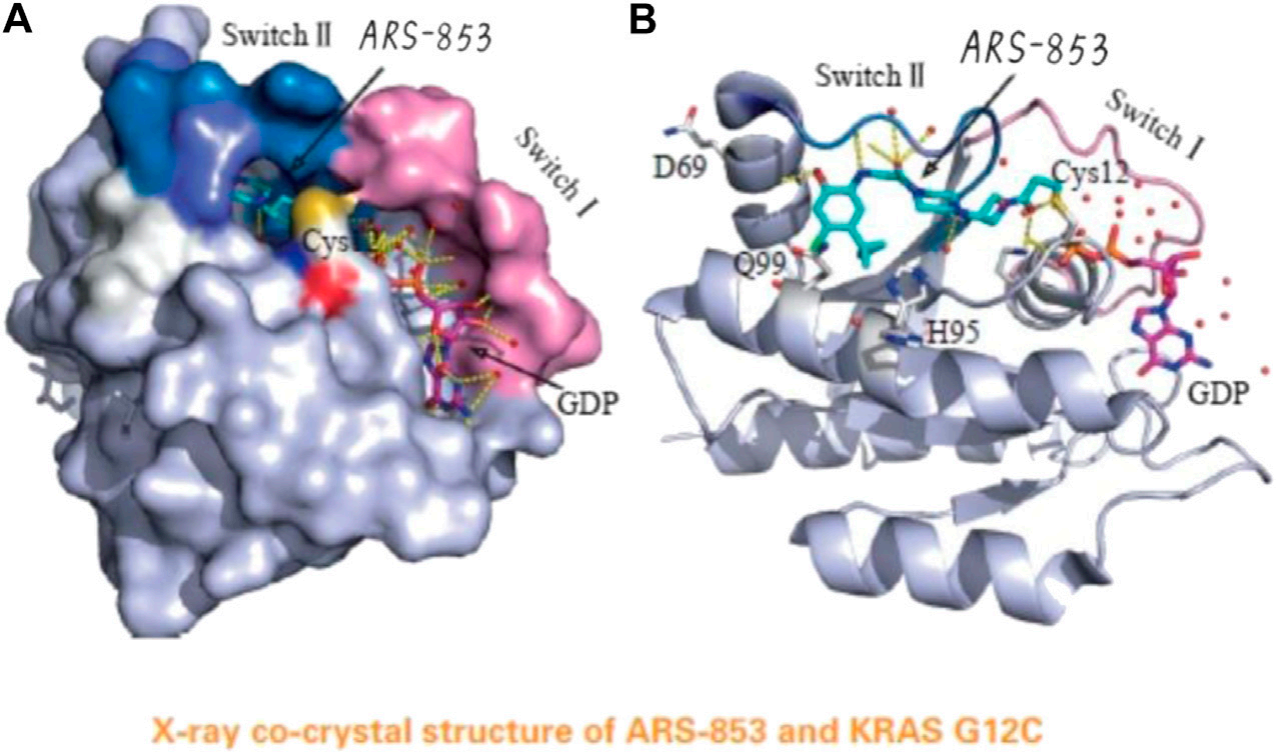
The acrylamide of ARS-853 can form covalent bond with 12 cysteine and extend to switch Ⅱ region, aromatic nucleus occupies hydrophobic region, the cyclopropyl group and the surrounding amino acids form a strong van der Waals force. (Reprinted by permission from Progrss in Pharmaceutical Sciences [14]).

**FIGURE 5:**
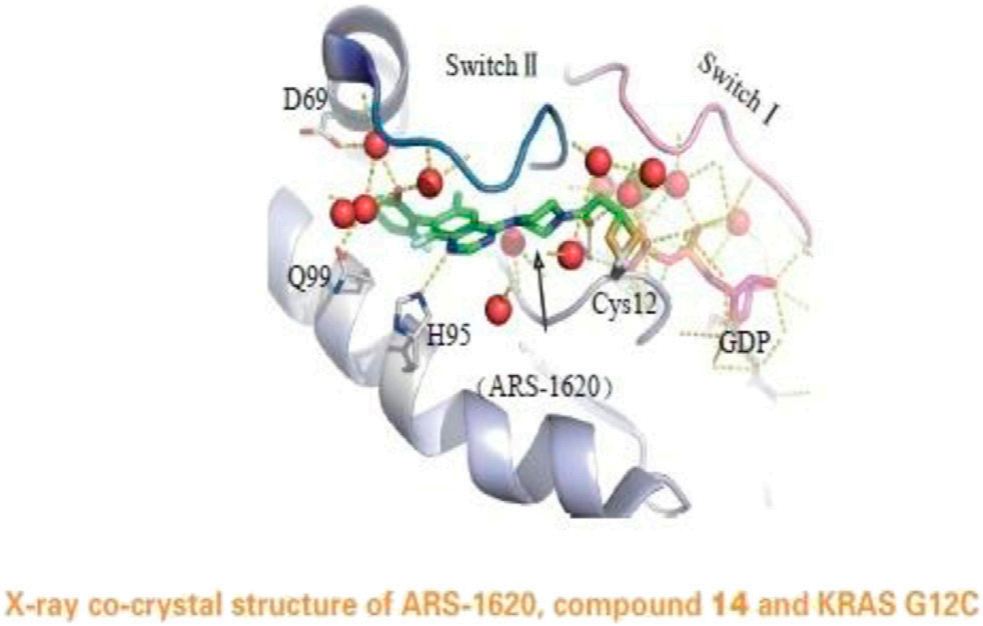
X-ray co-crystal structure of ARS-1620, compound 14 and KRAS G12C. (Reprinted by permission from Progrss in Pharmaceutical Sciences [14]).

**FIGURE 6:**
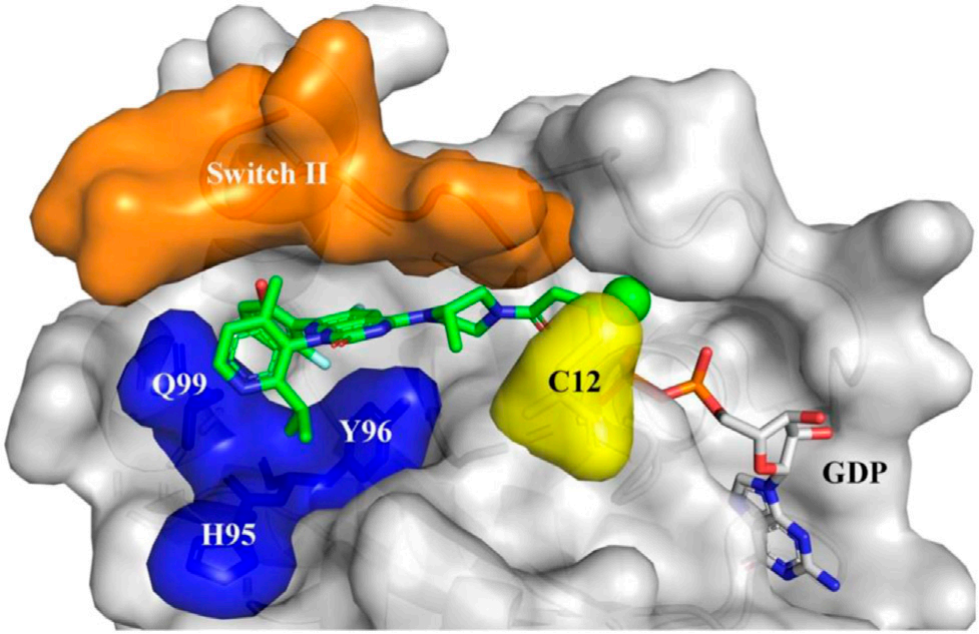
Sotorasib (AMG510) binding to KRAS-G12C protein. Orange: Switch II domain; Yellow: C12 residual; Blue: A cryptic pocket composed of H95, Y96, and Q99. AMG510 (green) and GDP (gray) are shown in sticks. (Reprinted by permission from Acta Pharmaceutica Sinica [20]).

**FIGURE 7:**
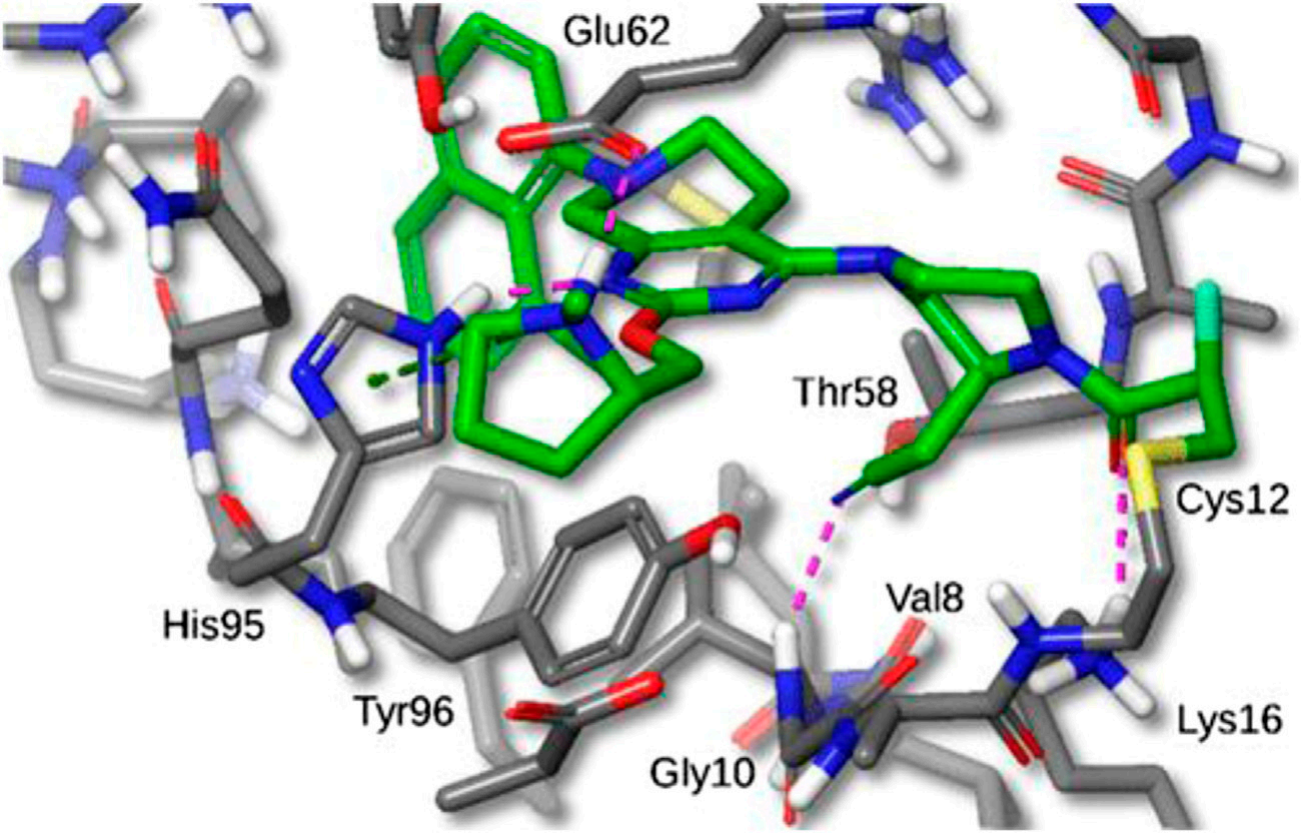
X-ray crystal structure of MRTX849 bound to KRASG12C with 1.94 Å resolution, hydrogens added for clarity. (Reprinted by permission from [30], further permissions related to the material excerpted should be directed to the ACS).

**FIGURE 8:**
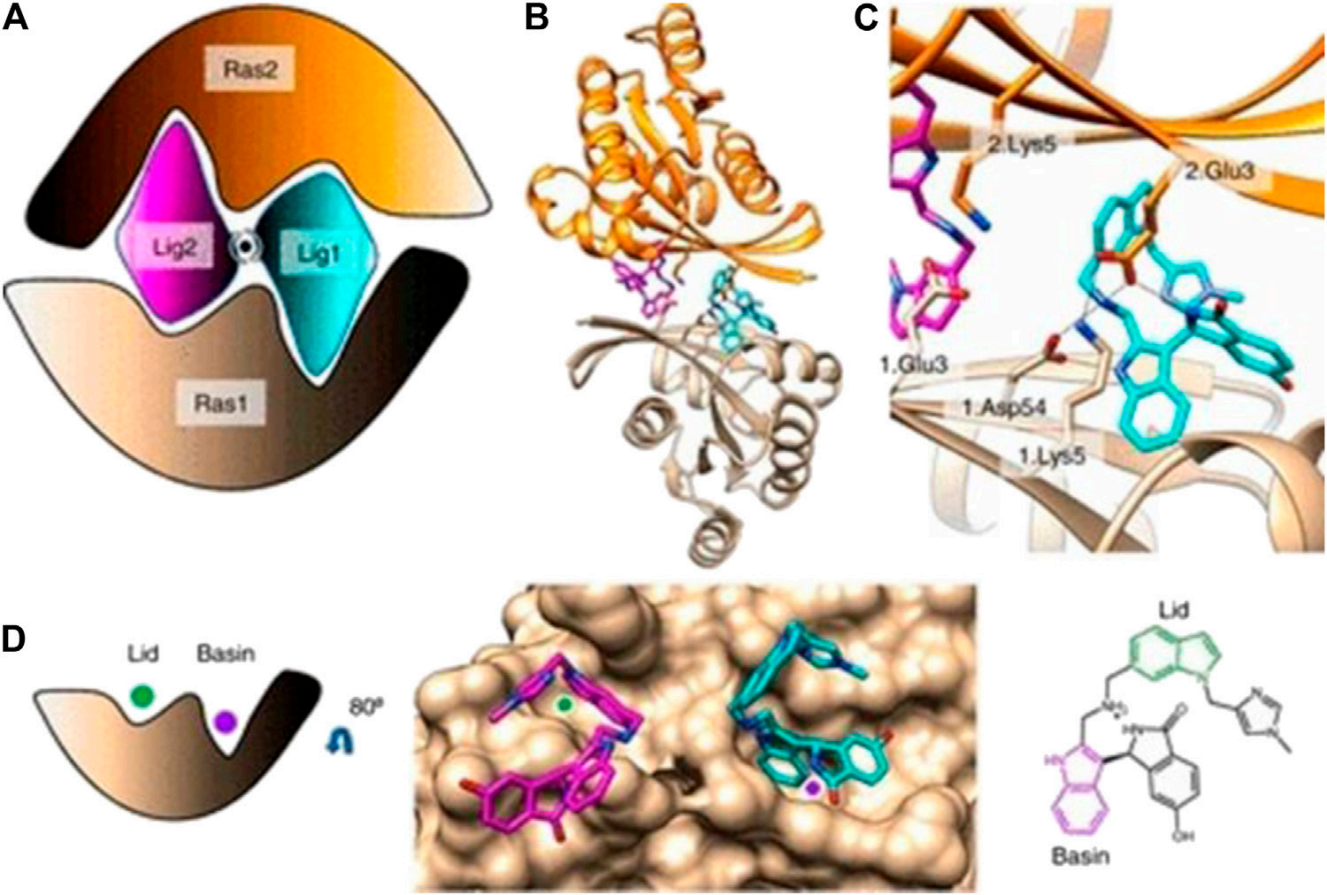
**(A)** Cartoon of BI-2852 KRAS dimer. Lig1 (cyan) and Lig2 (magenta) interact with both Ras1 (beige) and Ras2 (orange). **(B)** KRAS (ribbons) and key side chains are shown. **(C)** Zoom-in of the binding site: salt–bridge interactions shown (dashed lines). **(D)** Lid (green) and Basin (purple) are indicated on Ras1. Both Lig1 and Lig2 are shown on Ras1. The indole rings, colored green and purple, engage the Lid and Basin, respectively. (Reprinted by permission from Proc Natl Acad Sci U S A [33]).

The sources for the figures have been added to the reference list, and the reference numbering has been updated to accommodate this. The new references are appear below.

The authors apologize for this error and state that authorization has been obtained from the author of each picture, and this does not change the scientific conclusions of the article in any way. The original article has been updated.
